# Mechanical Properties Enhancement of the Au-Cu-Al Alloys via Phase Constitution Manipulation

**DOI:** 10.3390/ma14113122

**Published:** 2021-06-07

**Authors:** Kang-Wei Goo, Wan-Ting Chiu, Ayano Toriyabe, Masahiro Homma, Akira Umise, Masaki Tahara, Kenji Goto, Takumi Sannomiya, Hideki Hosoda

**Affiliations:** 1 Institute of Innovative Research (IIR), Tokyo Institute of Technology, 4259 Nagatsuta, Midori–ku, Yokohama 226–8503, Japan; chiu.w.aa@m.titech.ac.jp (W.-T.C.); toriyabe.a.aa@m.titech.ac.jp (A.T.); umise.a.aa@m.titech.ac.jp (A.U.); tahara.m.aa@m.titech.ac.jp (M.T.); 2 School of Materials and Chemical Technology, Tokyo Institute of Technology, 4259 Nagatsuta, Midori–ku, Yokohama 226–8503, Japan; homma.m.aa@m.titech.ac.jp (M.H.); sannomiya.t.aa@m.titech.ac.jp (T.S.); 3 TANAKA Kikinzoku Kogyo K.K., 28 Suzukawa, Isehara 259–1146, Japan; k-goto@ml.tanaka.co.jp

**Keywords:** α-phase, Au-Cu-Al system, fcc annealing twin, martensite phase, mechanical properties, microstructure, reflectance analysis

## Abstract

To enhance the mechanical properties (e.g., strength and elongation) of the face-centered cubic (fcc) α-phase in the Au-Cu-Al system, this study focused on the introduction of the martensite phase (doubled B19 (DB19) crystal structure of Au_2_CuAl) via the manipulation of alloy compositions. Fundamental evaluations, such as microstructure observations, phase identifications, thermal analysis, tensile behavior examinations, and reflectance analysis, have been conducted. The presence of fcc annealing twins was observed in both the optical microscope (OM) and the scanning electron microscope (SEM) images. Both strength and elongation of the alloys were greatly promoted while the DB19 martensite phase was introduced into the alloys. Amongst all the prepared specimens, the 47Au41Cu12Al and the 44Au44Cu12Al alloys performed the optimized mechanical properties. The enhancement of strength and ductility in these two alloys was achieved while the stress plateau was observed during the tensile deformation. A plot of the ultimate tensile strength (UTS) against fracture strain was constructed to illustrate the effects of the introduction of the DB19 martensite phase on the mechanical properties of the alloys. Regardless of the manipulation of the alloy compositions and the introduction of the DB19 martensite phase, the reflectance stayed almost identical to pure Au.

## 1. Introduction

Gold-copper-aluminum (Au-Cu-Al) alloy was developed for jewelry and biomedical applications due to its unique aesthetics and high biocompatibility [1,2]. In addition to the aforementioned aesthetics and biocompatibility issue, materials that contain heavy elements, such as Au, are preferred for medical applications due to their high X-ray contrast [3]. The α-phase, which is the (Au, Cu) terminal solid solution in the Au-Cu-Al system, possesses a face-centered cubic (fcc) crystal structure [4]. It is also well known that the integration of the fcc elements could develop the alloy into a medium and/or high entropy alloy (MEA and/or HEA) [5,6]. Furthermore, Al, which is also in a fcc crystal structure, was further introduced into the binary Au-Cu alloy in this work. Hence, the ternary Au-Cu-Al system could be considered as preliminary research for the promising MEA and/or HEA, which perform good mechanical properties.

To realize the Au-Cu-Al alloy for practical use, it is crucial to enhance the mechanical properties, such as strength and ductility, of the aforementioned fcc α-phase. Firstly, it is well known that the addition of a second phase could enhance the mechanical strength of a material due to precipitation hardening and/or dispersion hardening [7]. Secondly, a thermo-elastic martensite phase (doubled B19 (DB19) crystal structure of Au_2_CuAl) of the Au-Cu-Al system could undergo variant re-orientation (twin deformation of martensite) as external stress is applied to the material [8]. When the alloy undergoes deformation, the martensitic re-orientation could release the stress concentration [9], thus suppressing the crack formation in the alloy and increasing the elongation of the alloy by twin deformation. The enhancement of elongation is known as the twin-induced plasticity (TWIP) effect, which is also found in steel [10]. Finally, since the DB19 martensite plays the role of obstacle for the movement of dislocations [11], an increase of strength by suppressing the local inhomogeneous deformation and work hardening is foreseen.

Based on these premises, the introduction of the DB19 martensite phase into the abovementioned fcc α-phase alloy was expected to enhance the overall strength and elongation of this ternary Au-Cu-Al alloy system. It is necessary to mention that the DB19 martensite phase would be transformed from the parent β-phase (bcc-based L2_1_ crystal structure) when the temperature is below the reverse martensitic transformation start temperature (*A*_s_) [12]. This study, therefore, referred to the α+β region in the Au-Cu-Al alloy isothermal phase diagram at 773 K [13] for specimen fabrications.

The objective of this study was, thus, to introduce the DB19 martensite phase into the fcc α-phase of Au-Cu-Al alloy via the manipulation of Au and Cu concentrations. In this work, it was found that the DB19 martensite phase was successfully inserted into the fcc α-phase alloy in the ternary Au-Cu-Al system, resulting in the alloys with promoted mechanical properties. In addition, both the introduction of the second phase (i.e., the DB19 martensite phase) into the fcc α-phase alloy and the composition manipulation did not deviate the surface color and tinge of the alloys from the desired color of pure Au.

## 2. Materials and Methods

Au (99.99%), Cu (99.99%), and Al (99.99%) elements were used as raw materials for the fabrications of the Au-Cu-Al alloys. In the Au-Cu-Al system, the parent β-phase (bcc L2_1_ crystal structure) was transformed to the DB19 martensite while the temperature was below the reverse martensitic transformation start temperature (*A*_s_) [12]. Thus, by referring to the α+β region in the isothermal phase diagram at 773 K [13], four specimens with different nominal compositions were prepared. The nominal compositions of the specimens and the abbreviations of the specimens are shown in Table 1. The Al element in these alloys was kept at 12 at. % to assure that the stability of the alloy was the same for all specimens [14].

Materials in the total weight of 5 g were weighted based on the element percentage in Table 1 and the alloys were arc-melted by a non-consumable tungsten electrode in the Ar atmosphere. The weight losses of the specimens after arc-melting were confirmed to be less than 0.6%, as shown in Table 1. The alloys were then hot-forged at 873 K for 6 h under an Ar atmosphere to obtain a disk-shaped specimen with diameters of around 20 mm and thickness of approximately 2 mm. The disk-shaped specimens were cut into specific shapes for the sample analysis. Mechanical polishing was conducted thereafter for cleaning the ingot surface, and homogenization was carried out at 773 K for 1 h followed by quenching in iced water. Grinding and polishing for each analysis were conducted for the following measurements.

Optical microscope (OM; VHX-7000, Keyence, Osaka, Japan) and scanning electron microscope (SEM; S-4300SE, HITACHI, Tokyo, Japan) were utilized for the observation of microstructures and the determination of the grain sizes.

The phase constitutions were identified by using the X-ray diffractometer (XRD; X’Pert PRO, PANalytical, Malvern, UK) at an ambient temperature of 293 K (±3 K). Specimens were scanned from 20° to 90° with the CuK_α_ radiation (40 mA, 45 kV, λ = 0.15405 nm) at a scan rate of 0.042° s^−1^. A standard silicon plate was used as a reference for the calibration of the external system error.

The phase transformation temperatures were determined by the differential scanning calorimeters (DSC; DSC-60 Plus, Shimadzu Corporation, Kyoto, Japan) under an Ar atmosphere for the prevention of the oxidation reaction of the specimens. The temperature range was between 223 K and 523 K at the scan rate of 10 K/min and all the specimens were subjected to 2 scanning cycles. A standard alumina powder (Al_2_O_3_) in the identical weight with the specimens was used as a reference.

Tensile tests were performed 3 times for each specimen by using the universal testing machine (Autograph AG-Xplus 10 kN, Shimadzu Corporation, Kyoto, Japan) at an ambient temperature of 293 K (±3 K) and the strain rate was controlled at 8.3 × 10^−4^ s^−1^. The specimen size was 10 mm in length, 1 mm in width, and 0.3 mm in thickness. The cross-sections of the fracture surface were also observed by using the aforementioned SEM.

It is known that the surface color and tinge vary with the compositions and phase constitutions of the alloys [15,16]; therefore, a reflectance analysis was further conducted. Prior to the reflectance analysis, the surface of the specimens was ground and polished down to 0.05 μm alumina polishing suspension until a mirror-like surface finish was obtained. The examinations were performed by a spectrometer (C10083MD, Hamamatsu Photonics, Hamamatsu, Japan), which was equipped with a tungsten-halogen light source and a reflection sensor (HL-2000-HP and R400-7-UV-VIS, respectively, Ocean Optics, Orlando, FL, USA). The wavelength used was from 400 nm to 800 nm. A thick Al film deposited on glass was used for the reference. The result of the Al reference was corrected by theoretical reflectance. Pure Au specimen was also polished to an identical surface condition and was measured for comparisons with the Au-Cu-Al alloys.

## 3. Results and Discussion

### 3.1. Microstructure Observations

**Figure 1:**
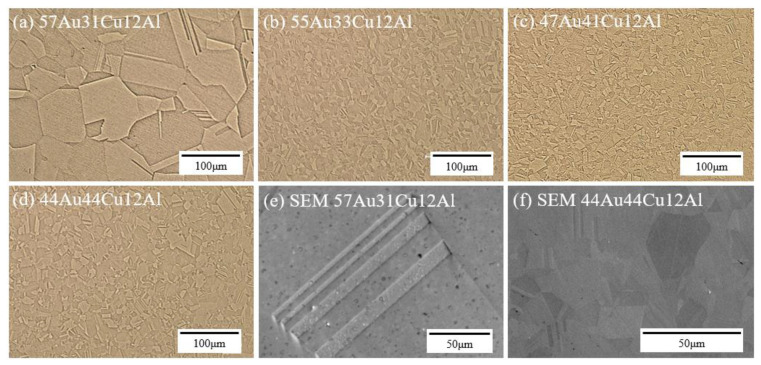
OM images of (**a**) 57Au31Cu12Al, (**b**) 55Au33Cu12Al, (**c**) 47Au41Cu12Al, and (**d**) 44Au44Cu12Al alloys as well as SEM images of (**e**) 57Au31Cu12Al and the (**f**) 44Au44Cu12Al alloys.

The preliminary microstructure observations of the specimens were conducted by an OM, as shown in Figure 1a–d. The grain boundaries could be seen clearly in all the OM images, therefore, the average grain size of each specimen was calculated based on the OM images by the common linear intercept method. The average grain sizes were 59, 15, 17, and 19 μm for (a) 57Au31Cu12Al, (b) 55Au33Cu12Al, (c) 47Au41Cu12Al, and (d) 44Au44Cu12Al alloys, respectively. It was deduced that the relatively large grain size of the (a) 57Au31Cu12Al alloy could be contributed by the absence of the DB19 martensite phase. More details are discussed in the following subsections.

Note that the presence of the fcc annealing twins, which are often formed in fcc materials under certain thermal treatments [17], were observed in the OM images of all alloys in Figure 1a–d. To reveal the fcc annealing twins clearly, SEM images of the 57Au31Cu12Al and the 44Au44Cu12Al alloys, which exhibit zoomed-in images of the fcc annealing twins, are shown in Figure 1e,f. The existence of fcc annealing twins indicates that the stacking fault energy (SFE) of the fcc α-phase could be low [18]. The occurrence of the fcc annealing twins was expected to cause planar slip during the tensile test [19]. Without the presence of annealing twins, the fcc α-phase alloy is expected to have high elongation since annealing twins lead to the aggregation of the dislocations [20]. This would be further discussed in the subsection of mechanical properties.

### 3.2. Phase Constitutions

**Figure 2:**
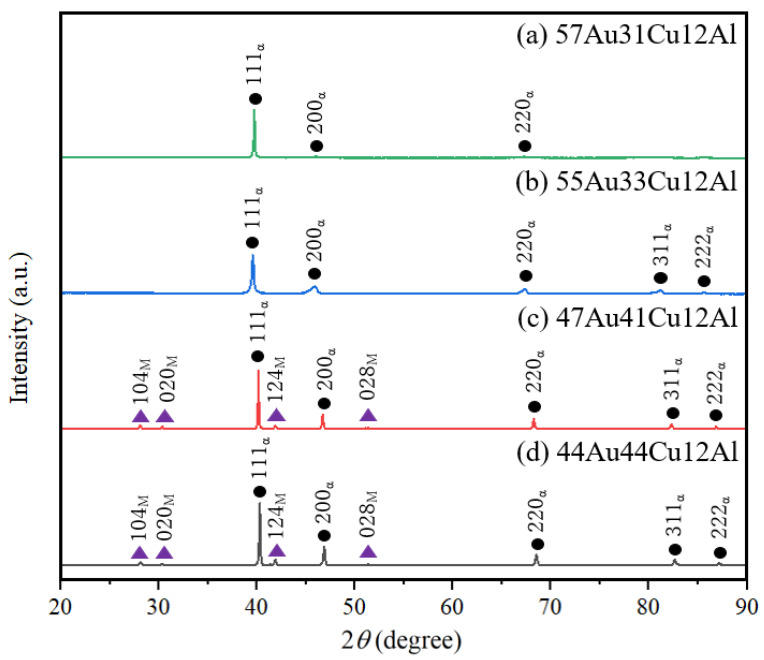
X-ray diffraction patterns of (**a**) 57Au31Cu12Al, (**b**) 55Au33Cu12Al, (**c**) 47Au41Cu12Al, and (**d**) 44Au44Cu12Al alloys at 293 K (±3 K). (The subscript of α indicates the α-phase and the subscript of M suggests the DB19 martensite phase).

Phase constitutions of the specimens were identified by the X-ray diffraction analysis at 293 K (±3 K), as shown in Figure 2. According to the X-ray diffraction patterns, the characteristic peaks of the α-phase (solid black circle symbols) were clearly observed in all the specimens. In addition, the DB19 martensite phase (solid violet triangle symbols) was further observed in the (c) 47Au41Cu12Al and the (d) 44Au44Cu12Al alloys. It could thus be deduced that the DB19 martensite phase was successfully introduced into the α-phase in the (c) 47Au41Cu12Al and the (d) 44Au44Cu12Al alloys [12]. The grain refinement of the (c) 47Au41Cu12Al and the (d) 44Au44Cu12Al alloys in Figure 1 could be attributed to the presence of the secondary DB19 martensite phase. It was thus speculated that the relatively small grains of the (b) 55Au33Cu12Al alloy could also be attributed to the limited deposition of the DB19 martensite phase, which might go beyond the detection limit of the XRD condition employed.

### 3.3. Thermal Analysis

**Figure 3:**
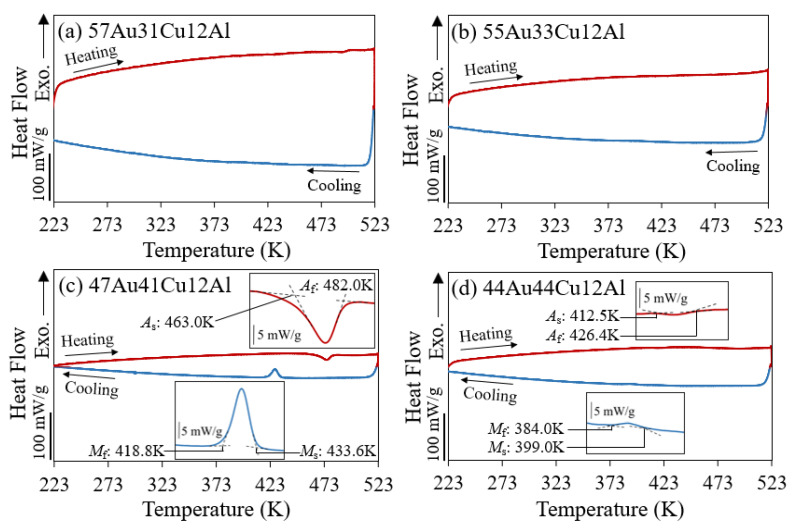
DSC curves of (**a**) 57Au31Cu12Al, (**b**) 55Au33Cu12Al, (**c**) 47Au41Cu12Al, and (**d**) 44Au44Cu12Al alloys. The inserted figures indicate the enlarged transformation peaks. *A*_s_, *A*_f_, *M*_s_, and *M*_f_ indicate the austenite (reverse martensite) transformation start temperature, austenite transformation finish temperature, martensite transformation start temperature, and martensite transformation finish temperature, respectively.

The results of the thermal analysis via DSC are shown in Figure 3, revealing the transformation temperatures of the alloys. Judging from the heating and cooling curves, the phase transformation temperatures were observed in the (c) 47Au41Cu12Al and the (d) 44Au44Cu12Al alloys in Figure 3c,d. On the other hand, no phase transformation was found in the (a) 57Au31Cu12Al and the (b) 55Au33Cu12Al alloys in Figure 3a,b.

In accordance with the X-ray diffraction observations (Figure 2), it could be deduced that the phase transformation temperatures in the thermal analysis results were related to the presence of DB19 martensite phase. More analysis is necessary in order to identify the cause of the peaks presented. It may be caused by either martensite-austenite phase transition or diffusion-controlled martensitic-like transition [21]. Both phase transformations were found during heating and cooling in those alloys possessing the DB19 martensite phase at 293 K (±3 K) (i.e., the (c) 47Au41Cu12Al and the (d) 44Au44Cu12Al alloys in Figure 2). On the contrary, there was an absence of the phase transformations in those alloys, which only consisted of the single α-phase (i.e., the (a) 57Au31Cu12Al and the (b) 55Au33Cu12Al alloys in Figure 2). It thus could be concluded that the results of the thermal analysis (Figure 3) agreed well with those of the X-ray diffraction measurements (Figure 2).

A higher volume fraction of the DB19 martensite phase was reported in the (c) 47Au41Cu12Al alloy than that of the (d) 44Au44Cu12Al alloy according to the isothermal phase diagram [13] and was found in the X-ray diffraction patterns (Figure 2). Relatively large exothermic and endothermic peaks in the (c) 47Au41Cu12Al alloy (Figure 3c) were detected. In addition, it was further found that both the phase transformation temperatures in cooling and heating curves of the (c) 47Au41Cu12Al alloy were higher than that of the (d) 44Au44Cu12Al alloy in Figure 3. This could be attributed to the difference in the internal stress caused by the different amounts of the secondary DB19 martensite phase between these two alloys since the intensity of the DB19 martensite peaks in the X-ray diffraction results (Figure 2) were different from each other.

### 3.4. Mechanical Properties

**Figure 4:**
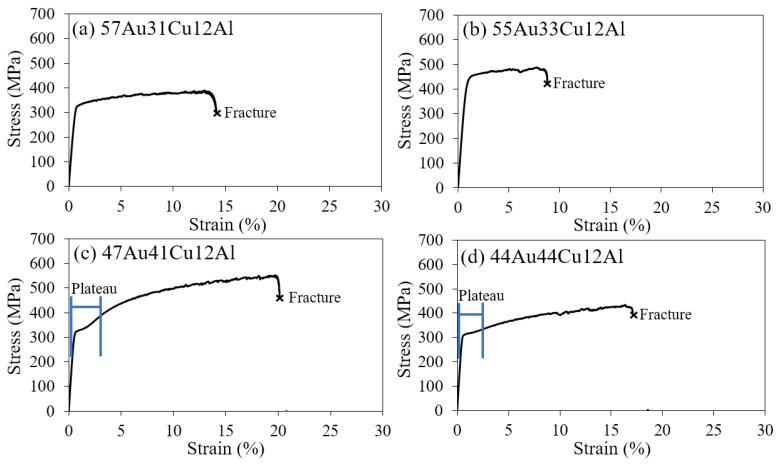
Stress–strain curves of the tensile tests of (**a**) 57Au31Cu12Al, (**b**) 55Au33Cu12Al, (**c**) 47Au41Cu12Al, and (**d**) 44Au44Cu12Al alloys at 293 K (±3 K). The cross symbols indicate the fracture of the specimens.

Figure 4 shows the stress–strain curves (S-S curves) of tensile tests conducted in ambient at 293 K (±3 K). All the specimens showed certain work hardening rates in Figure 4; this could be brought by the presence of the planar slip deformation by the fcc annealing twins that were observed in the OM and SEM images in all the specimens, as mentioned in the microstructural observation subsection. Besides, it was observed that since the specimen of the (a) 57Au31Cu12Al alloy possessed a relatively large average grain size, its yielding stress was low compared to the specimen of the (b) 55Au33Cu12Al alloy.

On the other hand, the specimens containing both the α-phase and the DB19 martensite phase (i.e., the (c) 47Au41Cu12Al and the (d) 44Au44Cu12Al alloys in Figure 2) performed high elongation, while the alloys possessing the single α-phase (i.e., the (a) 57Au31Cu12Al and the (b) 55Au33Cu12Al alloys in Figure 2) exhibited relatively low ductility. The high elongation of the (c) 47Au41Cu12Al and the (d) 44Au44Cu12Al alloys could be ascribed to the re-orientation of the DB19 martensite phase, which could enhance the ductility of the specimen [8,9,10,11]. The stress plateau for the re-orientation of the martensite phase was indicated by the blue lines in Figure 4c,d. It was obvious that the re-orientation of the martensite could be found in the (c) 47Au41Cu12Al and the (d) 44Au44Cu12Al alloys, while the clear stress plateau was not found in the (a) 57Au31Cu12Al and the (b) 55Au33Cu12Al alloys.

Furthermore, the martensitic re-orientation plateau is more distinct in the (c) 47Au41Cu12Al alloy than that of the (d) 44Au44Cu12Al alloy since it is confirmed that the (c) 47Au41Cu12Al alloy consists of a higher volume fraction of the DB19 martensite phase than that of the (d) 44Au44Cu12Al alloy, as explained in the X-ray diffraction and thermal analysis subsections. This statement is also consistent with the expected phase volume fractions between the fcc α-phase and the parent β-phase, which were deduced from the isothermal phase diagram [13]. The expected parent β-phase volume fraction of the (c) 47Au41Cu12Al alloy is around 13%, which is higher than that of the 10% of the (d) 44Au44Cu12Al alloy. Hence, after phase transformation to low temperature (i.e., 293 K of the operation temperature), the volume fraction of the DB19 martensite phase in the (c) 47Au41Cu12Al and the (d) 44Au44Cu12Al alloys should be at approximately 13% and 10%, respectively.

It is worth mentioning that the X-ray diffraction patterns, thermal analysis, and tensile tests agreed well with each other. Note that the strain of the plateau was relatively large as the phase volume fraction of the DB19 martensite phase was larger in the case of the (c) 47Au41Cu12Al alloy than that of the (d) 44Au44Cu12Al alloy. It is also necessary to mention that since the (c) 47Au41Cu12Al and the (d) 44Au44Cu12Al alloys consisted of both the fcc annealing twins and DB19 martensite phase, the strain of the plateau could be an outcome of a combination of two different deformation mechanisms, such as the planar slip by fcc annealing twins and the martensitic re-orientation of from the DB19 martensite phase. Based on the aforementioned results, enhancements of both strength and elongation were achieved in the (c) 47Au41Cu12Al and the (d) 44Au44Cu12Al alloys (Figure 4).

**Figure 5:**
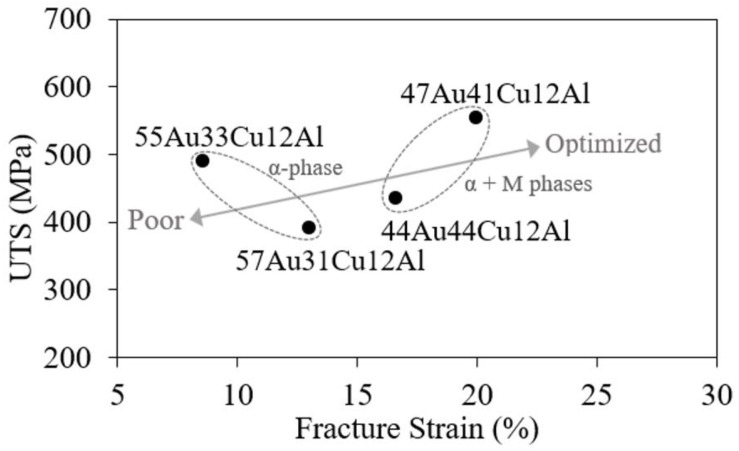
The relationship between the UTS and the fraction strain of 57Au31Cu12Al, 55Au33Cu12Al, 47Au41Cu12Al, and 44Au44Cu12Al alloys. (The α and M indicate the α-phase and the DB19 martensite phase, respectively).

The Hall-Petch relationship was not applied in this study as there was a difference in the composition and deformation mechanism (i.e., planar slip deformation and martensitic re-orientation) among the alloys. To reveal the trend of the mechanical properties, a plot of UTS as a function of fracture strain is shown in Figure 5. Alloys that consisted of the single α-phase (i.e., the (a) 57Au31Cu12Al and the (b) 55Au33Cu12Al alloys) possessed a relatively low fracture strain compared to the alloys with both the α-phase and the DB19 martensite phase (i.e., the (c) 47Au41Cu12Al and the (d) 44Au44Cu12Al alloys). The improved ductility could be ascribed to the re-orientation of the DB19 martensite phase, which was explained in the previous subsection. Owing to the presence of the martensite variant re-orientation, the (c) 47Au41Cu12Al alloy demonstrates the highest elongation and strength among all the alloys in this work accordingly. The arrow in Figure 5 points out the optimized alloys, which both possessed high strength and high elongation. It can thus be concluded that the strength and elongation of the fcc α-phase alloy in the Au-Cu-Al system could be improved by the introduction of the DB19 martensite phase.

**Figure 6:**
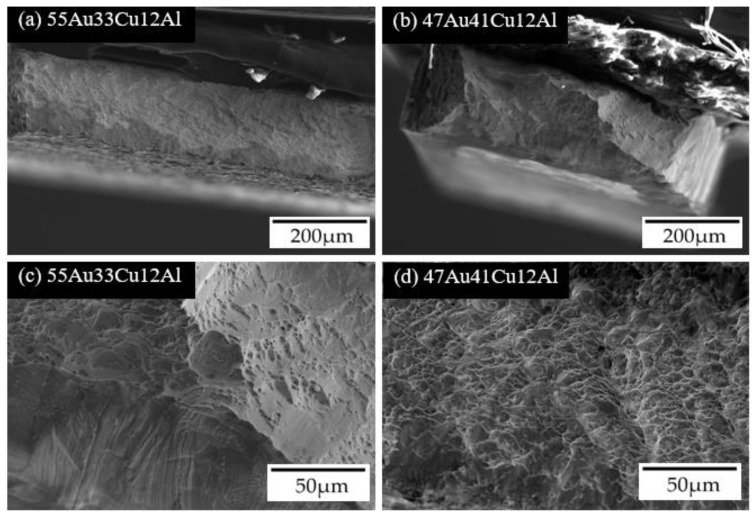
The cross-section SEM images and the zoomed-in images of (**a**,**c**) 55Au33Cu12Al and (**b**,**d**) 47Au41Cu12Al alloys.

The alloys of the 55Au33Cu12Al and the 47Au41Cu12Al, which performed the lowest and the highest ductility, respectively, were chosen for the observations of the cross-sections of the fracture surface for the purpose of understanding the difference of fracture mechanisms between the single α-phase alloys (i.e., the 55Au33Cu12Al alloy) and the dual α-phase + DB19 martensite phase alloys (i.e., the 47Au41Cu12Al alloy). During the tensile test, stress concentration accumulated at the fcc annealing twins and the single α-phase alloys (i.e., the 57Au31Cu12Al and the 55Au33Cu12Al alloys) would undergo the localization deformation. Different from the aforementioned alloys, specimens, which possessed the dual α-phase + DB19 martensite phase (i.e., the 47Au41Cu12Al and the 44Au44Cu12Al alloys), have higher ductility due to the re-orientation of the DB19 martensite phase during the tensile test. It was obvious that the cross-section of the fracture surface of the 55Au33Cu12Al alloy (Figure 6a,c) and the 47Au41Cu12Al alloy (Figure 6b,d) showed typical brittle and ductile surface morphologies, respectively [22]. The dimples, which were clearly observed in Figure 6d in a relatively large amount, indicated that the 47Au41Cu12Al alloy underwent ductile fracture.

Concerning the relationships among microstructures, phase constitutions, and mechanical properties, further research is being conducted by our research group. More information regarding the ternary Au-Cu-Al alloy system and its high-order system will be published in our future articles.

### 3.5. Reflectance Analysis

The applications of the Au-Cu-Al system are not limited to biomedical devices, as mentioned in the introduction section, as it could also be applied to our daily life. Since the color and tinge of the Au-Cu-Al alloys could be manipulated via fine-tuning of alloy composition [15,16], it could also be applied to pieces of jewelry and dental braces as well. Therefore, the color analysis was further conducted in this study by referring to their reflectance in the range of the visible light wavelength (400–800 nm).

**Figure 7:**
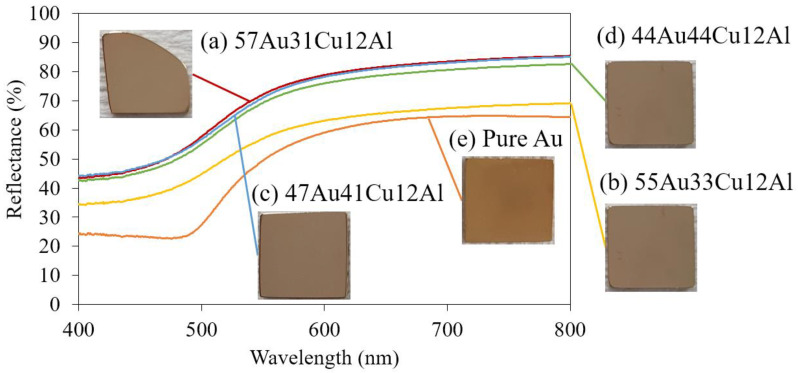
Reflectance analysis results of (**a**) 57Au31Cu12Al, (**b**) 55Au33Cu12Al, (**c**) 47Au41Cu12Al, and (**d**) 44Au44Cu12Al alloys as well as the (**e**) pure Au.

Figure 7 shows the reflectance of each specimen as a function of the wavelength and the inserted photos are the corresponding alloys, which were ground and polished to a mirror-like finish. While observing the specimens with naked eyes, all four alloys displayed similar color, which was in the range of pale orange-yellow, while pure Au seemed to have a comparatively distinct yellow tinge (refer to the specimen photos in Figure 7). This observation is consistent with the reflectance analysis illustrated in Figure 7, where the slope of all the curves of four specimens started to decrease in the range of 550–600 nm, indicating that all specimens performed in a similar color, which was in the yellow range. This could be concluded that the performance of the color for the aesthetic property did not vary, while the compositions of Au and Cu were manipulated.

It is also necessary to mention that there was a presence of a minor DB19 martensite phase in the 47Au41Cu12Al and the 44Au44Cu12Al alloys (Figure 2c,d), while no significant difference was found in the reflectance between the single α-phase and the dual α-phase + DB19 martensite phase. This signified both of these phases in these alloys have similar reflectance characteristics, regardless of their differences in composition and crystal structure. On the other hand, it was deduced that the influence of grain size could be limited since the reflectance curve of the 57Au31Cu12Al alloy was almost identical to the 47Au41Cu12Al and the 44Au44Cu12Al alloys. The difference in absolute reflectance of the specimens could be attributed to the dissimilarity in surface roughness; even the polishing procedures were kept identical to the greatest extent.

## 4. Conclusions

The Au-Cu-Al alloys with different compositions were prepared and the fundamental analysis, such as microstructure observations, phase identifications, mechanical property evaluations, and the color analysis, were carried out in this study. The important findings are listed in the following bullets.

1. The fcc annealing twins were observed in the microstructure observation of all specimens, indicating that the stacking fault energy of the fcc α-phase in these alloys could be low.
2. The desired dual-phase in the 47Au41Cu12Al and the 44Au44Cu12Al alloys, which were composed of the fcc α-phase and double B19 (DB19) martensite phase, were obtained via the manipulation of their compositions.
3. The mechanical properties, such as strength and elongation, were greatly promoted in the 47Au41Cu12Al alloy, followed by the elongation enhancement of the 44Au44Cu12Al alloy owing to the insertion of the DB19 martensite phase.
4. Stress plateau in the stress–strain curves were observed in the 2 highest elongation specimens (i.e., the 47Au41Cu12Al and the 44Au44Cu12Al alloys), which were composed of both the fcc α-phase and the DB19 martensite phase.
5. The color remained almost intact while the DB19 martensite phase was successfully introduced into the fcc α-phase by varying the Au and Cu concentrations of the alloys for the enhancement of strength and ductility. In addition, according to the color analysis, the color of specimens in this study was close to pure Au.
6. The usage of the noble metal Au in the Au-Cu-Al alloys was greatly reduced, while the strength and ductility were greatly improved, and the surface tinge remained almost intact.

## Figures and Tables

**Table 1:** Nominal compositions of the fabricated Au-Cu-Al alloys.

Specimen	Nominal Composition (at.%)			Abbreviation	Mass Loss after Arc-Melting (%)
	Au	Cu	Al		
(a)	57	31	12	57Au31Cu12Al	0.02
(b)	55	33	12	55Au33Cu12Al	0.01
(c)	47	41	12	47Au41Cu12Al	0.52
(d)	44	44	12	44Au44Cu12Al	0.46

## Data Availability

The study did not report any data.
